# Evaluation of Fat Accumulation and Adipokine Production during the Long-Term Adipogenic Differentiation of Porcine Intramuscular Preadipocytes and Study of the Influence of Immunobiotics

**DOI:** 10.3390/cells9071715

**Published:** 2020-07-17

**Authors:** Asuka Tada, AKM Humayun Kober, Md. Aminul Islam, Manami Igata, Michihiro Takagi, Masahiko Suzuki, Hisashi Aso, Wakako Ikeda-Ohtsubo, Kazutoyo Yoda, Kenji Miyazawa, Fang He, Hideki Takahashi, Julio Villena, Haruki Kitazawa

**Affiliations:** 1 Food and Feed Immunology Group, Laboratory of Animal Products Chemistry, Graduate School of Agricultural Science, Tohoku University, Sendai 980-8572, Japan; a-tada@morinagamilk.co.jp (A.T.); humayuna2002@yahoo.com (A.H.K.); aminul.vmed@bau.edu.bd (M.A.I.); manamiigata@gmail.com (M.I.); takagimichihiro@gmail.com (M.T.); masah-suzuki@itoen.co.jp (M.S.); wakako.ohtsubo.a7@tohoku.ac.jp (W.I.-O.); 2 Livestock Immunology Unit, International Education and Research Center for Food and Agricultural Immunology (CFAI), Graduate School of Agricultural Science, Tohoku University, Sendai 980-8572, Japan; asosan@tohoku.ac.jp; 3 Department of Dairy and Poultry Science, Chittagong Veterinary and Animal Sciences University, Chittagong 4225, Bangladesh; 4 Department of Medicine, Faculty of Veterinary Science, Bangladesh Agricultural University, Mymensingh 2202, Bangladesh; 5 Cell Biology Laboratory, Graduate School of Agricultural Science, Tohoku University, Sendai 980-8572, Japan; 6 Technical Research Laboratory, Takanashi Milk Products Co., Ltd., Yokohama Kanagawa 241-0023, Japan; k-yoda@takanashi-milk.co.jp (K.Y.); ke-miyazawa@takanashi-milk.co.jp (K.M.); KA-HOU@takanashi-milk.co.jp (F.H.); 7 Laboratory of Plant Pathology, Graduate School of Agricultural Science, Tohoku University, Sendai 980-8572, Japan; hideki.takahashi.d5@tohoku.ac.jp; 8 Plant Immunology Unit, International Education and Research Center for Food Agricultural Immunology, Graduate School of Agricultural Science, Tohoku University, Sendai 980-8572, Japan; 9 Laboratory of Immunobiotechnology, Reference Centre for Lactobacilli, (CERELA-CONICET), Tucuman 4000, Argentina

**Keywords:** porcine intramuscular adipocyte, long-term adipogenic differentiation (LTAD), inflammation, adipokines, fat accumulation, immunobiotics

## Abstract

The degree of fat accumulation and adipokine production are two major indicators of obesity that are correlated with increased adipose tissue mass and chronic inflammatory responses. Adipocytes have been considered effector cells for the inflammatory responses due to their capacity to express Toll-like receptors (TLRs). In this study, we evaluated the degree of fat accumulation and adipokine production in porcine intramuscular preadipocyte (PIP) cells maintained for in vitro differentiation over a long period without or with stimulation of either TNF-α or TLR2-, TLR3-, or TLR4-ligands. The cytosolic fat accumulation was measured by liquid chromatography and the expression of adipokines (CCL2, IL-6, IL-8 and IL-10) were quantified by RT-qPCR and ELISA at several time points (0 to 20 days) of PIP cells differentiation. Long-term adipogenic differentiation (LTAD) induced a progressive fat accumulation in the adipocytes over time. Activation of TLR3 and TLR4 resulted in an increased rate of fat accumulation into the adipocytes over the LTAD. The production of CCL2, IL-8 and IL-6 were significantly increased in unstimulated adipocytes during the LTAD, while IL-10 expression remained stable over the studied period. An increasing trend of adiponectin and leptin production was also observed during the LTAD. On the other hand, the stimulation of adipocytes with TLRs agonists or TNF-α resulted in an increasing trend of CCL2, IL-6 and IL-8 production while IL-10 remained stable in all four treatments during the LTAD. We also examined the influences of several immunoregulatory probiotic strains (immunobiotics) on the modulation of the fat accumulation and adipokine production using supernatants of immunobiotic-treated intestinal immune cells and the LTAD of PIP cells. Immunobiotics have shown a strain-specific ability to modulate the fat accumulation and adipokine production, and differentiation of adipocytes. Here, we expanded the utility and potential application of our in vitro PIP cells model by evaluating an LTAD period (20 days) in order to elucidate further insights of chronic inflammatory pathobiology of adipocytes associated with obesity as well as to explore the prospects of immunomodulatory intervention for obesity such as immunobiotics.

## 1. Introduction

Obesity is a global public health concern having a high socio-economic impact. According to the World Health Organization, globally there are about 1.9 billion overweight adults, 650 million of whom are obese, and worldwide obesity has nearly tripled since 1975 until 2016 [1]. Obesity is characterized by increased adipose tissue mass in the whole body, which is associated with abnormal or excessive fat accumulation in the adipocytes along with subclinical chronic inflammatory responses [2]. However, the consequence of obesity-associated metabolic disorders mostly depends on whether adipose depot is expanded by the increase of adipocyte size (hypertrophy), or by the formation of new adipocytes from precursors (hyperplasia) [3]. The balance of hypertrophic expansion of existing adipocytes and the development of new adipocytes through adipogenesis within the individual has a profound impact on metabolism. Healthy white adipose tissue expansion is achieved by the recruitment and differentiation of adipose precursor cells rather than the increment of fat in mature adipocytes [4]. However, in obesity, the excessive hypertrophied adipocytes have been associated with metabolic abnormalities such as type 2 diabetes, hypertension, cardiovascular diseases, hepatic steatosis, musculoskeletal disorders, metabolic disorders and cancer [5,6]. Adipocyte hypertrophy has also been associated with elevated lipolysis [7], increased inflammatory cytokines secretion [8], and reduced production of anti-inflammatory adipokines such as leptin [8] and adiponectin [9]. On the other hand, adipogenic expansion through preadipocytes differentiation can offset the negative impact of metabolic disorders [3]. Modulation of adipogenic differentiation by the immune system has been considered as an adaptive response that is essential for healthy remodeling of adipose tissue and its normal function [10]. Therefore, a deep understanding of the cellular and molecular changes of fat accumulation and inflammatory responses over the long-term adipogenic differentiation (LTAD) of preadipocytes is of great interest.

There are two major classes of adipose tissue in the mammalian body: the white adipose tissue that is specialized to store excess energy as fat, and the brown adipose tissue, which has a remarkable ability to dissipate excess energy from substrate (lipids and glucose) oxidation directly as heat [11]. On the other hand, adipocytes can be found in different anatomical locations (subcutaneous, visceral, intermuscular, intramuscular or bone depots) having different physiological and pathological properties [12]. Adipocytes interjecting themselves between and among skeletal muscle fibers are called intramuscular adipocytes and in meat animals, they are termed overall as a marbling fat [13]. There is considerable knowledge on preadipocyte proliferation, differentiation into mature cells, adipocyte ability to conduct lipid metabolism or produce adipokines in normal conditions and in the context of abnormal metabolism. Most of these studies have been performed in visceral adipocytes while less is known about the cellular and molecular changes of intramuscular adipocytes in both physiological and pathological conditions [14]. In order to study the immunobiology and adipogenesis of porcine intramuscular white adipose tissue in vitro, we have previously established a clonal porcine intramuscular preadipocyte (PIP) line from the Musculus longissimus thoracis muscle tissue of Duroc pigs [15], which have bred to produce marbling muscle [16]. We reported that adipogenesis in the PIP cell line can be induced by a combination of octanoate and oleate in the presence of dexamethasone and insulin, which results in the production of functionally mature adipocytes [15]. Therefore, this cell line has been used for the study of adipogenesis. Moreover, we also demonstrated that PIP cells and mature adipocytes derived from them after a short differentiation period are able to respond to acute challenges with cytokines or microbial products by triggering inflammatory responses [17]. Therefore, the PIP cell model is considered relevant for studying adipogenesis and inflammation of adipocytes in the porcine host and its impact on high-quality meat production.

On the other hand, attention has focused on the composition of fat within human skeletal muscle as determinants of insulin resistance and involvement in the metabolic syndrome [18,19]. Then, considering the anatomical, physiological and immunological similarities between pigs and humans [14], it is tempting to speculate that the PIP cell line could also be used as a human model. We speculated that the characterization of the immunobiology and adipogenesis of PIP cells under a sustained rather than an acute inflammatory environment could help to position this cell line as a useful laboratory tool for metabolic and immunological studies of adipocytes in the context of obesity, as well as therapeutic alternatives to mitigate its adverse effects.

Innate immune receptors, in particular the Toll-like receptors (TLRs) family, are likely to be involved in obesity-associated inflammatory signaling as the over-expression of TLR2 and TLR4 has been observed in the adipose tissue of obese individuals [20,21]. Activation of TLRs stimulate adipocytes to secrete adipokines, which contribute to the development of local and systemic inflammatory responses [22,23]. Expressions of TLRs including TLR-2, -3 and -4, and their ligand responsiveness have been demonstrated in adipocytes of human [24] and mouse [25] origins. In this regard, we previously demonstrated that nine members of TLR family (TLR-1-9) are expressed in PIP cells as well as in differentiated mature porcine adipocytes [17]. In a recent study, we also demonstrated the acute activation of TLR-2, -3 and -4 in porcine adipocytes by their ligand stimulation resulted in overexpression of several cytokines and chemokines including interleukin 8 (IL-8), IL-6 and the C-C motif chemokine ligand 2 (CCL2) [26], which were reported to be linked with obesity-related inflammatory responses in adipocytes [27]. In addition, TNF-α has been implicated as a mediator in the induction of insulin resistance and adipose tissue inflammation [28]. In vitro studies suggested that TNF-α affects glucose homeostasis and fat accumulation in adipocytes, as well as their differentiation (reviewed in ref [29]). We have also demonstrated that TNF-α stimulation significantly affect the biology of PIP cells during in vitro differentiation for a period of four days [17,30].

Immunoregulatory probiotic bacteria or immunobiotics are known to be involved in the beneficial modulation of the host immune responses [31,32]. As adipocytes do not come in direct contact with orally administered immunobiotic bacteria, the cross-talk between immunobiotics and gut immune cells is believed to influence the adipocyte’s immunity [32,33]. In a previous study, our research group demonstrated that probiotic strains including *Lactobacillus rhamnosus* GG, *L. gasseri* TMC0356, and *L. rhamnosus* LA-2 were able to exert immunobiotic effects with significant reduction in the expression of proinflammatory cytokines and chemokines in adipocytes after an acute challenge with TNF-α [17]. Exploring the trend of immunobiotic-mediated changes in adipocytes over a longer period of differentiation and under a sustained inflammation would provide a better understanding of their potential benefits on the progressive fat accumulation and the chronic inflammatory responses of adipocytes.

The elucidation of the immunological regulators and the cellular and molecular mechanisms involved in the process of adipogenesis are of great interest in order to improve our understanding of the adipose tissue physiology and pathology as well as to develop new strategies to reduce their negative consequences in the obese host. In the present work, we investigated the effects of LTAD on the progressive fat accumulation and adipokines production in the porcine intramuscular adipocytes. We also studied whether immunobiotic strains are able to influence fat accumulation and/or inflammation during LTAD. This work constitutes a step forward to establish an in vitro model that could allow the study of the effects of sustained inflammation on the biology of adipocytes as well as the beneficial effect of immunobiotics in this context.

## 2. Materials and Methods

### 2.1. Cells and Culture Conditions

The PIP cell line, originally established by our group [15] was used in the present study. The culture condition and adipogenesis induction were performed according to the method described previously [17,33]. Briefly, the PIP cells were cultured in Dulbecco’s modified Eagle medium (DMEM, Gibco, Paiseley, Scotland, UK) with 10% fetal calf serum (FCS), 100 U/mL penicillin, and 100 mg/mL streptomycin as a growth medium by using 75 cm^2^ flask (BD Japan, Tokyo, Japan). The 4-day post-confluent PIP cells were washed with phosphate buffer saline (PBS), and stimulated with PBS containing 0.04% EDTA and kept in a CO_2_ incubator for 5 min with Trypsin buffer (0.04% EDTA, 0.02% trypsin in PBS). Cells were prepared at a density of 2.5 × 10^4^/cm^2^ and were induced to long-term adipogenesis (20 days) by adding a differentiation medium: DMEM containing 10% FBS, 50 ng/mL insulin (swine, Sigma), 0.25 μM dexamethasone (Sigma), 2 mM octanoate (Wako), 200 μM oleate (Ardorich, Milwaukee, WI, USA), 100 U/mL penicillin, and 100 μg/mL streptomycin. The medium was changed at every second day. The cells and culture supernatants were collected at day 0, 1, 2, 4, 8, 12, 16 and day 20 of differentiation for performing studies.

Antigen presenting cells (APCs) were isolated from porcine Peyer’s patches according to the method described in our previous publication [34]. Briefly, porcine Peyer´s patches were cut into small pieces and gently pressed through nylon mesh to prepare single immune cell suspensions. After several washes in complete RPMI medium, residual erythrocytes were lysed in 0.2% NaCl followed by a hypertonic rescue in 1.5% NaCl. Then, immune cells were fractionated by density gradient centrifugation using Lymphocyte Mammal (Cedarlane, Corbyville, ON, Canada) and the mononuclear cell suspension containing a mixed population of T, B and APCs was suspended in complete DMEM supplemented with 10% FCS, 50 U/mL penicillin and 50 μg/mL streptomycin (Nacalai Tseque, Kyoto, Japan). Finally, the APCs including macrophage and dendritic cells were separated by their ability to adhere to a glass plate.

### 2.2. Triglyceride Assay

Cytosolic triglyceride (TG) content of porcine adipocytes was analyzed using the TG assay kit (Wako Chemicals Inc., Virginia, USA) according to the manufacturer’s instruction. Briefly, cells were harvested by using a cell scraper and crushed by ultrasonic fragmentation followed by the treatment with 600 µL of lysis buffer (20 mm Tris, 1 mM EDTA). Then, 100 µL of lysate were used for protein assay, and the remaining volume was used for TG assay. Lipid extraction was performed according to Folch’s method [35]. In brief, 500 µL of Chloroform–methanol (2:1, *v*/*v*) were added to the lysate sample. The lysate samples were subjected for ultrasonic fragmentation then centrifuged at 15,000 rpm for 5 min at 4 °C. Then cellular protein content was measured by BCA (bicinchoninic acid) protein assay kit (Thermo Fisher Scientific, Inc., Waltham, MA, USA) and the TG data was normalized by protein concentration (µg/mg protein).

### 2.3. Oil red O and Hematoxylin Staining

For quantitative analysis of differentiation and cytosolic fat accumulation in the adipocytes, cultured cells were stained with Oil red O staining kit (GENMED Scientifics INC., Wilmington, DE, USA) according to the protocol described in previous publications [17,36]. In brief, cells were rinsed three times in PBS and then fixed in 10% (*v*/*v*) formalin for 30 min, then cells were dissolved in isopropanol for 1 min and then stained with Oil red O (Sigma-Aldrich, Tokyo, Japan) for 30 min at 37 °C. Thereafter, cells were washed twice with deionized water and stained with Mayer’s Hematoxylin solution (Merck, Darmstadt, Germany). The stained cells were then washed with deionized water and immersed in aqueous ammonia. Then, the cells were dried to examine and snapped the lipid droplet accumulation under the inverted tissue culture microscope (Olympus IX70). Photographs of stained cells were further analyzed for the size and number of cells by using the ImageJ software (National Institute of Health, Bethesda, MD, USA) according to the method described by Parlee et al. [36].

### 2.4. Fatty acid Analysis by Gas–Liquid Chromatography (GLC)

GLC-based fatty acid analysis of the adipocytes culture-supernatant was performed according to our previous publication [17]. In brief, the processed samples were converted to fatty acid methyl esters and analyzed using a Hitachi G-6000 gas chromatograph equipped with flame ionization detectors and a TC-70 column (0.25 mm × 60 m, GL Sciences Inc., Tokyo, Japan) in Helium carrier gas. The concentrations of three free fatty acids: palmitic acid, stearic acid and oleic acid released by the adipocyte were measured by GLC.

### 2.5. Induction of Fat Accumulation by SFAs Stimulation

Adipocytes were cultured at a density of 2.5 × 10^4^/cm^2^ in 6-well plates (BD Falcon, Tokyo, Japan). The 4-day-old confluent cells maintained in differentiation medium were stimulated by exogenous SFAs: palmitic acid, myristic acid and stearic acid (Sigma-Aldrich, Osaka, Japan) each with four different doses (0 as control, and 250, 25, and 2.5 μmol/L) and kept for the next 4 days. Both SFA treated and untreated cells were washed with PBS three times to remove the oleic acid left in the differentiation medium, and the samples were harvested for TG assay. The degree of fat accumulation into the adipocytes was quantified by TG assay kit as described earlier.

### 2.6. Induction of Inflammatory Responses during the Long-Term Adipogenic Differentiation (LTAD)

Inflammatory responses were induced in differentiated adipocytes by the stimulation with TNF-α and three chemical ligands: Pam3CSK4, poly(I:C) and LPS for the activation of TLR2, TLR3 and TLR4, respectively. Pam3CSK4 is a synthetic triacylated lipopeptide, poly(I:C) is a synthetic double-stranded RNA while LPS is purified from the cell-wall of Gram-negative bacteria. Preadipocytes were cultured at a density of 2.5 × 10^4^/cm^2^ in a six-well plate (BD Falcon, Tokyo, Japan) and 4-day post confluent PIP cells treated to induce their differentiation as described previously. The differentiated adipocytes were stimulated either by TNF-α (2.5 ng/mL), Pam3CSK4 (10 ng/mL), poly(I:C) (0.1 μg/mL) or LPS (0.1 μg/mL) for 12 h and kept for 20 days with a regular changing of culture media. In order to determine the dose of each stimulant, CCL2 expression was verified. The concentration of TLR ligands were chosen according to their ability to induce 5-fold increase of CCL2 expression. TNF-α concentration was chosen according to our previous study [17]. TNF-α (2.5 ng/mL) stimulation resulted in a 10-fold increase in the expression of CCL2 when compared to control. The mRNA and protein of four adipokines: CCL2, IL-6, IL-8, and IL-10 were determined by RT-qPCR and ELISA, respectively, as described below in Section 2.9 and Section 2.10.

### 2.7. Lactic Acid Bacteria Strains

Bacterial isolates were provided by the Takanashi Milk Product Company (Yokohama, Japan). Seven strains of lactic acid bacteria (LAB) were used in this study: *L. gasseri* TMC0356, *L. rhamnosus* GG, *L. rhamnosus* LA-2, *L. paracasei* TMC0409, *Streptococcus thermophilus* TMC1543, *Bifidobacterium bifidum* TMC3108 and *B. bifidum* TMC3115. Lactobacilli and bifidobacteria strains were grown in MRS (deMan-Rogosa-Sharp) medium (Difco, Detroit, MI, USA) for 16 h at 37 °C. *S. thermophilus* was grown in Elliker medium (Difco, Detroit, MI, USA) for 16 h at 37 °C. The cultured bacteria were washed with PBS and heated at 60 °C for 30 min. After washing twice with PBS, resuspended in DMEM (10% FCS, 1% streptomycin/penicillin) and adjusted 2.5 × 10^9^ cells/mL.

### 2.8. Effect of Immunobiotic Stimulations on the Fat Accumulation and Adipokines Production in Adipocytes

For the assessment of immunobiotic effect in terms of their potential to regulate fat accumulation and inflammatory responses of adipocytes, we accomplished two types of in vitro experiments. First, mononuclear cells isolated from porcine Peyer’s patches were stimulated with the different LAB strains. The immunocompetent cells were seeded in a 6-well cell culture plate (BD Falcon, 1 × 10^6^ cells/well) and stimulated with LAB (5 × 10^7^ cells/well) in 5% CO_2_ humidified atmosphere at 37 °C, for 24 h, in a complete RPMI 1640 medium (Sigma) supplemented with 2% fetal bovine serum (FCS). After stimulation, cell free supernatant (CFS) was collected for further experiments. Twenty-day-old differentiated mature porcine adipocytes were pre-stimulated for 48 h with CFS. In a second set of experiments, adipocytes (1 × 10^6^ cells/well) were directly stimulated with LAB strains (5 × 10^7^ cells/well) for 48 h.

### 2.9. Quantitative Real Time PCR

Total RNA was isolated from treated and untreated cells using TRIzol reagent (Invitrogen) according to our previous study [17]. The cDNAs were synthesized using a Quantitect reverse transcription (RT) kit (Qiagen, Tokyo, Japan) according to the manufacturer’s protocol. The RT-qPCR was performed with an Applied Biosystems Real-time PCR System 7300 (Applied Biosystems, Warrington, UK), using the Platinum SYBR Green qPCR SuperMix-UDG (uracil-DNA glycosylase) with ROX (6-carboxyl-X-rhodamine) (Invitrogen). The sequences of primers for CCL2, IL-6, IL-8, IL-10 and β-actin used for this study are available in our previous publications [17,34]. The PCR thermal cycling conditions were 5 min at 50 °C, followed by 5 min at 95 °C, and then 40 cycles of 15 s at 95 °C, 30 s at 60 °C, and 30 s at 72 °C. The reaction mixtures contained 2.5 μL of sample cDNA and 7.5 μL of master mix including the sense and antisense primers. Each reaction ran in duplicate and the expression of β-actin in each sample was used as a reference to normalize differences between samples and to calculate the normalized fold expression of target genes.

### 2.10. ELISA Assay

The protein concentration of IL-6, IL-8, IL-10, CCL2, leptin and adiponectin in the cell-free supernatant (100 μL) of adipocytes culture were measured by using commercially available ELISA kits: porcine IL-6, IL-8, and IL-10 ELISA kits (R&D Systems, Minneapolis, MN, USA); porcine CCL2 ELISA kit (Kingfisher Biotech, Inc. ST. Paul, MN, USA); pig leptin ELISA Kit (Wuhan Huamei Biotech Co., Ltd., Wuhan, China); and pig adiponectin ELISA Kit (Wuhan Huamei Biotech Co., Ltd., Wuhan, China). Estimation of the concentration of each protein was performed according to the manufacturers’ instructions. 

### 2.11. Statistical Analysis

All results were expressed as mean ± SD and are the average of three independent experiments. Relative indices were calculated as the ratio of fat deposition in adipocyte cells, and the values are normalized by common logarithmic transformation and confirmed as approximate values included significantly into normal distribution by the Kolmogorov–Smirnov test. Then, they were adjusted similarly to the mean of control groups. One-way analysis of variance (ANOVA) followed by Fisher’s least significant difference test was performed by the GLM procedure of the SAS program (Version 9.1) for the pairwise mean comparisons. Mean comparison of mRNA expression and protein concentrations between control (day 0) and several time points were performed by a repeated measures ANOVA procedure by using the PROC GLM model implemented in the SAS program considering the ‘day 0 measurement’ as a fixed factor.

## 3. Results

### 3.1. Fat Accumulation and Fatty Acid Production in Porcine Adipocytes during LTAD

**Figure 1:**
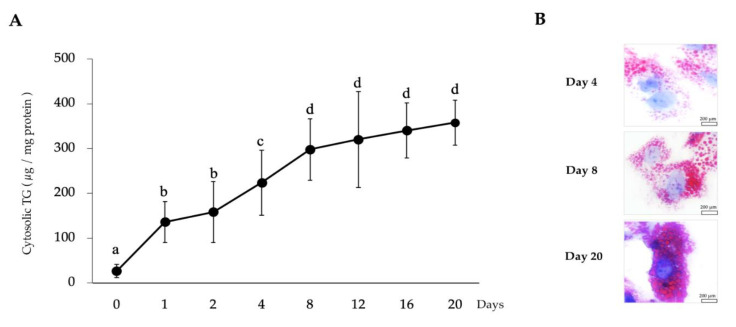
Long-term adipogenic differentiation mediated fat accumulation into the porcine intramuscular preadipocyte (PIP) in vitro culture. The PIP cells (2.5 × 10^4^/cm^2^) were kept in vitro culture for 20 days with differentiation medium, and cells were harvested at different time points to estimate the degree of cytosolic fat accumulation by using a triglyceride (TG) assay kit (**A**). Data shown are the mean ± SD of three independent experiments performed in triplicates. Different letters (a, b, c, and d) labeled upon the time points indicate significant differences (*p* < 0.05), while the same letter indicates no significant difference between the contrast pairs. Oil red O staining of adipocytes demonstrated the fat droplets red color while the nucleus takes a blue color when observed under microscope (**B**).

In order to investigate the effect of LTAD on the fat accumulation of porcine adipocytes, quantitative analysis of TG was performed. Continuous fat accumulation was observed over the 20 days of differentiation (Figure 1A). The cytosolic TG content was remarkably increased when PIP cells (before differentiation) were compared with adipocytes. Fat content was increased about 10-fold after 20 days of culture with the differentiation medium. Oil red O staining also confirmed the progressive accumulation of fat in the cytoplasm of adipocytes (Figure 1B). The lipid droplets within the adipocytes were increased in number and size in a time dependent manner (Figure 1B).

### 3.2. Size and Number of Adipocyte Counts during LTAD

**Figure 2:**
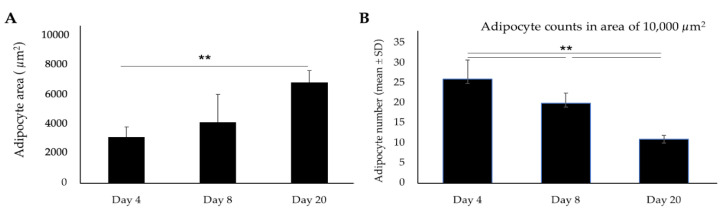
Measurement of proliferation and size of porcine adipocyte during long-term adipogenic differentiation. (**A**) The average area (size) of the adipocytes. (**B**) Average count of Oil red O stained (live) adipocytes. Images of stained adipocytes were analyzed for size and number of cells using ImageJ software. Data shown are the mean ± SD of three independent experiments performed in triplicates. The asterisks (**) indicated statistical differences of adipocyte number and size when compared with Day 4 at the significance level of *p* < 0.01.

The size of the adipocytes was also progressively increased over the longer period of differentiation (Figure 2A). The average number of live adipocytes (as estimated in a stained area of 10,000 μm^2^) was significantly decreased over time (Figure 2B).

### 3.3. Release of Free Fatty Acids from Porcine Adipocytes during LTAD

**Figure 3:**
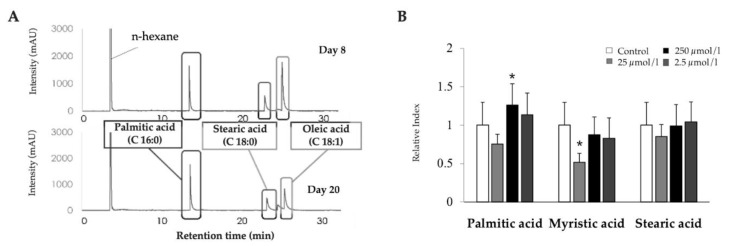
Long-term adipogenic differentiation (LTAD) mediated release of free fatty acids (FFA) from adipocytes, and the influence of saturated fatty acids (SFA) in fat accumulation. The concentrations of three FFA: Palmitic acid, Stearic acid and Oleic acid were measured in the differentiated adipocyte by gas–liquid chromatography (**A**). The 4-day-old confluent adipocytes (2.5 × 10^4^ /cm^2^) were challenged with three different concentrations (as of 250, 25, and 2.5 µmol/l) of each of three SFA for 4 days followed by quantifying the fat accumulation by a TG assay kit (**B**). The asterisk (*) indicated statistical differences of fat accumulation of SFA treated adipocyte when compared to untreated control with significance level of *p* < 0.05.

The release of free fatty acids by adipocytes during LTAD was also investigated (Figure 3A). Since the fat accumulation reached its relative stationary phase at day 8 and continued until day 20, the FFA contents of adipocytes were measured at day 8 and 20 of differentiation. There were no remarkable differences of palmitic acid (C 16:0), stearic acid (C 18:0) and oleic acid (C 18:1) contents in the culture supernatant measured between days 8 and 20 (Figure 3A). However, the stearic acid: oleic acid ratio was changed by the LTAD.

We also evaluated the ability of FFA to modify fat accumulation in adipocytes. For this purpose, adipocytes were stimulated with different concentrations of palmitic acid, myristic acid or stearic acid (0.1, 0.01 or 0.001 µg/mL) and the fat accumulation was evaluated (Figure 3B). Palmitic acid stimulation significantly increased the fat accumulation in adipocytes at a concentration lower than 0.01 µg/mL while myristic acid significantly reduced fat accumulation when used at a concentration of 0.1 µg/mL (Figure 3B). However, no effect was observed for any dose for the stearic acid (Figure 3B).

### 3.4. Adipokine Expression Dynamics in Adipocytes during LTAD

**Figure 4:**
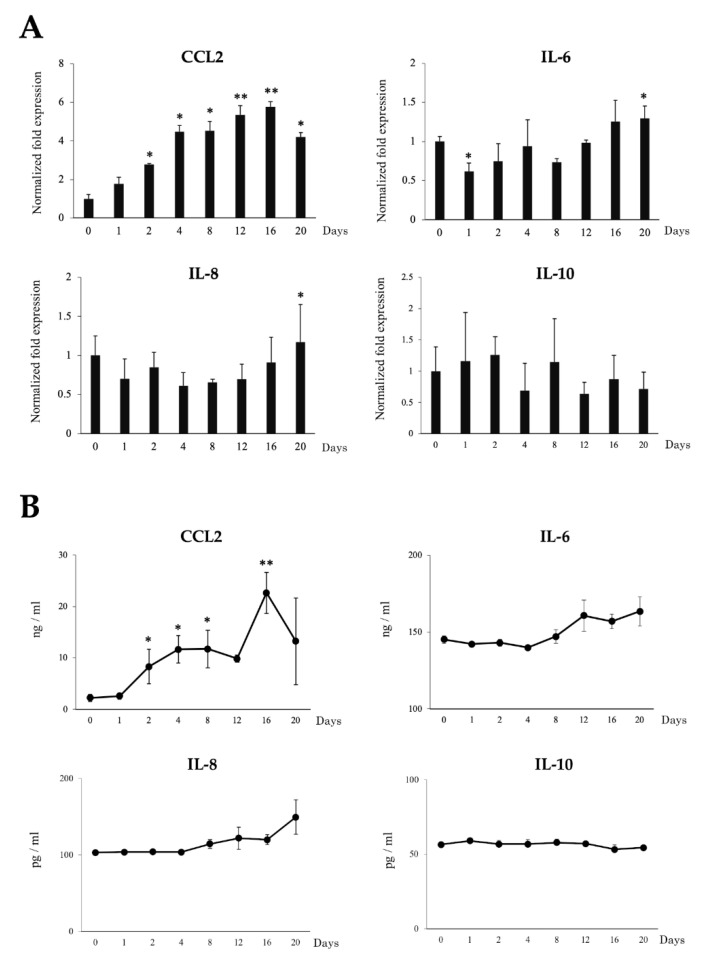
Long-term adipogenic differentiation mediated expression dynamics of adipokines in the adipocytes without any exogenous inflammatory triggers. The mRNA expression (**A**) and protein concentration (**B**) of CCL2, IL-6, IL-8 and IL-10 in adipocytes over the 20 days of culture as measured by RT-qPCR and ELISA, respectively. Data shown are the mean ± SD of three independent experiments performed in triplicates. The asterisks: (*) and (**) indicated statistical differences when compared with day 0 at the significance levels of *p* < 0.05 and *p* < 0.01, respectively.

**Figure 5:**
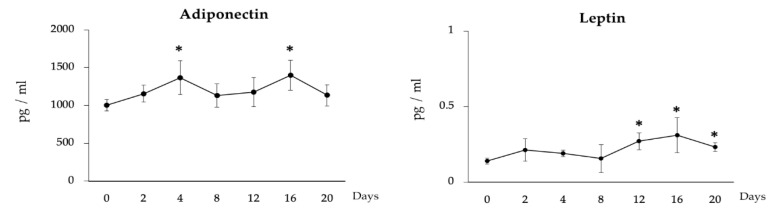
Long-term adipogenic differentiation mediated secretion of adiponectin and leptin in adipocytes. The ELISA-based quantification of adiponectin and leptin concentration in adipocytes as measured over the 20 days of culture. Data shown are the mean ± SD of three independent experiments performed in triplicates. The asterisk (*) indicated the statistical differences of protein concentration when compared with day 0, at the significance level of *p* < 0.05.

To examine the effects of LTAD in production of immune mediators, mRNA expression and protein concentration of four adipokines IL-6, IL-8, IL-10 and CCL2 were examined during the 20 days of adipogenesis (Figure 4). The mRNA expression of *CCL2* increased progressively during LTDA while the expression of *IL-6* and *IL-8* were significantly increased only at the end of the studied period. On the other hand, no significant changes were observed for *IL-10* mRNA (Figure 4A). The protein concentration of the adipokines evaluated changed in a similar fashion as observed for the mRNA levels (Figure 4B). Furthermore, the secretion of leptin and adiponectin by the adipocytes was measured by ELISA during the 20 days of differentiation (Figure 5). Significantly augmented levels were observed at days 4 and 16 for adiponectin, while leptin production was significantly increased after day 12 of adipogenesis.

### 3.5. Effect of TNF-α and TLR Ligands on Fat Accumulation in Adipocytes during LTAD

**Figure 6:**
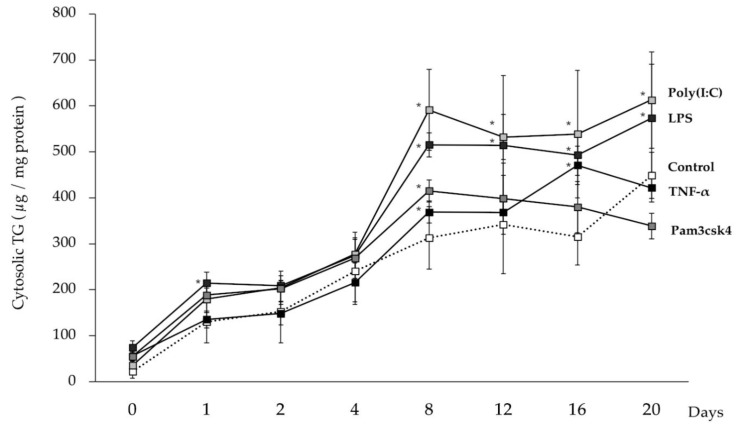
Regulation of fat accumulation in adipocytes by the stimulation of immune receptors. Adipocytes were stimulated either by TNF-α (2.5 ng/mL) or Pam3csk4 (10 ng/mL) or poly(I:C) (0.1 μg/mL) or LPS (0.1 μg/mL) for 12 h and kept for 20 days with regular changing culture media. Pam3CSK4, poly(I:C), and LPS represent the ligands for TLR2, TLR3 and TLR4, respectively. Data shown are the average ± SD of 3 independent experiments performed in triplicates. The asterisk (*) indicated the statistical difference between each treated group cells and the untreated control of corresponding time points, at the significance levels of *p* < 0.05.

In order to evaluate the effect of TNF-α and TLR ligands on the fat accumulation of porcine adipocytes, the cytosolic TG content was estimated. Progressive fat accumulation was observed in TNF-α and TLR-ligands stimulated adipocytes as well as in unstimulated cells (Figure 6). Interestingly, fat deposition rate was accelerated in adipocytes challenged with TLR3 or TLR4 ligands since significantly higher levels of TG were found from day 8 until the end of the studied period when compared to controls. On the other hand, TNF-α and TLR2 ligand stimulation did not result in significant changes in fat deposition as compared to control adipocytes at the end of the study period (Figure 6).

### 3.6. Effect of TNF-α on the Adipokine Production in Adipocytes during LTAD

**Figure 7:**
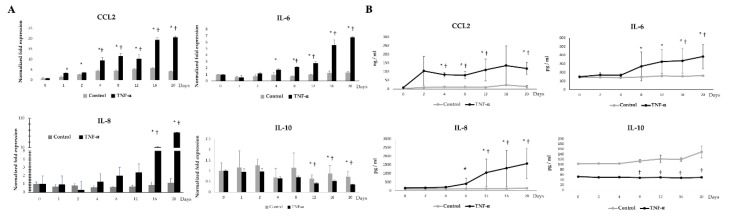
TNF-α mediated adipokine production in adipocytes. The mRNA expressions (**A**) and protein concentration (**B**) of CCL2, IL-6, IL-8, IL-10 in the adipocytes at different time points of 20 days of differentiation. Data shown are the mean ± SD of three independent experiments performed in triplicates. The ‘*’ indicated statistical differences when compared with day 0, and ‘†’ indicated statistical differences when compared with control group at the same time point, both at the significance level of *p* < 0.05.

We next aimed to evaluate whether the stimulation with TNF-α was able to change the expressions of four adipokines in porcine adipocytes during LTAD. As observed in non-stimulated control adipocytes, TNF-α-challenged adipocytes showed a progressive augment in CCL2 expression and protein concentration (Figure 7). However, adipocytes under TNF-α stimulation had significantly higher levels of CCL2 mRNA (Figure 7A) and protein (Figure 7B) when compared to unchallenged controls. In fact, *CCL2* expression in controls was up-regulated four-fold at day 2 while in TNF-α stimulated adipocytes this adipokine increased more than 20-fold when compared to basal levels. Similarly, both mRNA and protein levels of IL-8 and IL-6 were increased from day 8 (Figure 7) and their values were significantly higher when compared to unchallenged adipocytes. Interestingly, mRNA levels for *IL-10* were significantly reduced in TNF-α-challenged adipocytes already from day 8 onwards. In addition, a slight but not significant decrease in IL-10 protein concentration was observed after 20 days of LTAD (Figure 7B).

### 3.7. Effects of TLR2 on the Adipokine Production in Adipocytes during LTAD

**Figure 8:**
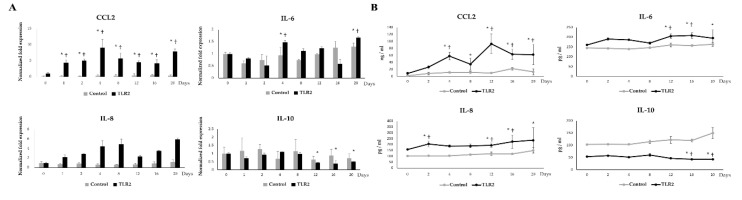
TLR2 mediated adipokine production in adipocytes. The figure displayed the TLR2 mediated mRNA expression (**A**) and protein concentration (**B**) of CCL2, IL-6, IL-8, IL-10 in the adipocytes at different time points of 20 days of adipogenesis. Data shown are the mean ± SD of three independent experiments performed in triplicates. The ‘*’ indicated statistical differences when compared with day 0, and ‘†’ indicated statistical differences when compared with control group at the same time point, both at the significance level of *p* < 0.05.

In order to investigate the effects of TLR2 activation during LTAD, adipocytes were challenged with Pam3CSK4 or LPS, respectively (Figure 8). TLR2 activation resulted in a fluctuant but significant increase of CCL2, IL-8 and IL-6 mRNA expression and protein concentrations during the period of adipogenesis studied (Figure 8A,B). TLR2-activated adipocytes also showed a significant reduction of IL-10 mRNA (Figure 8A) and protein (Figure 8B) levels from day 12 onward.

### 3.8. Effects of TLR4 on the Adipokine Production in Adipocytes during LTAD

**Figure 9:**
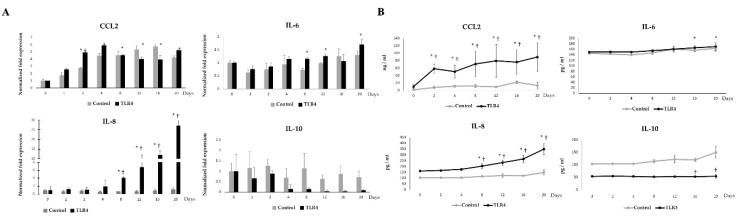
TLR4 mediated adipokine production in adipocytes. The figure displayed the TLR4 mediated mRNA expression (**A**) and protein concentration (**B**) of CCL2, IL-6, IL-8, IL-10 in the adipocytes at different time points of 20 days of adipogenesis. Data shown are the mean ± SD of three independent experiments performed in triplicates. The ‘*’ indicated statistical differences when compared with day 0, and ‘†’ indicated statistical differences when compared with control group at the same time point, both at the significance level of *p* < 0.05.

In order to investigate the effects of TLR4 activation during LTAD, adipocytes were challenged with Pam3CSK4 or LPS, respectively (Figure 9). TLR4 activation also resulted in a significant up-regulation of CCL2, IL-6 and IL-8 (Figure 9A,B). Particularly, the TLR4-activated adipocytes showed a sustained increase in CCL2 mRNA (Figure 9A) and protein concentration (Figure 9B) from day 1 of LTAD, while IL-8 and IL-6 were up-regulated from days 8 and 12, respectively (Figure 9A,B). On the contrary, both mRNA expression (Figure 9A) and protein concentration (Figure 9B) of IL-10 were significantly reduced after TLR4 activation.

### 3.9. Effect of TLR3 on the Adipokine Production in Adipocytes during LTAD

**Figure 10:**
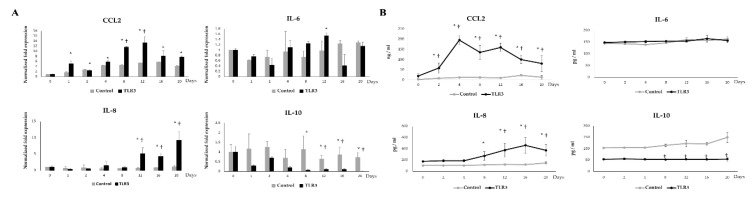
TLR3 mediated adipokine production in adipocytes. The mRNA expressions (**A**) and protein concentration (**B**) of CCL2, IL-6, IL-8, IL-10 in the adipocytes at different time points of 20 days of differentiation. Data shown are the mean ±SD of 3 independent experiments performed in triplicates. The ‘*’ indicated statistical differences when compared with day 0, and ‘†’ indicated statistical differences when compared with the control group at the same time point, both at the significance level of *p* < 0.05.

We also investigated the effect of the activation of TLR3 during LTAD. The stimulation of adipocytes with the TLR3 ligand poly(I:C) significantly up-regulated the mRNA expression and the protein concentration of the chemokine CCL2 when compared with before treatment time point (day 0) as well as untreated unchallenged control cells at corresponding time points (Figure 10A,B). In addition, poly(I:C) induced an increase in IL-8 mRNA levels (Figure 10A) and protein concentration (Figure 10B). The mRNA expression of IL-6 was increased at 12 h post poly(I:C) treated adipocytes (Figure 10A), while no significant variations in IL-6 protein level was observed when compared to the basal level (Figure 10B). The expression of IL-10 was significantly reduced from day 8 onwards when compared to before treatment (day 0), as well as corresponding control at the same time point (Figure 10A), while IL-10 protein concentration showed no differences when compared with before treatment time point (day 0) but significant difference as compared to the unchallenged control cells at day 8, 12, 16 and 20 (Figure 10B).

### 3.10. Immunobiotic Mediated Regulation of Fat Accumulation and Adipokine Production in Adipocytes during LTAD

**Figure 11:**
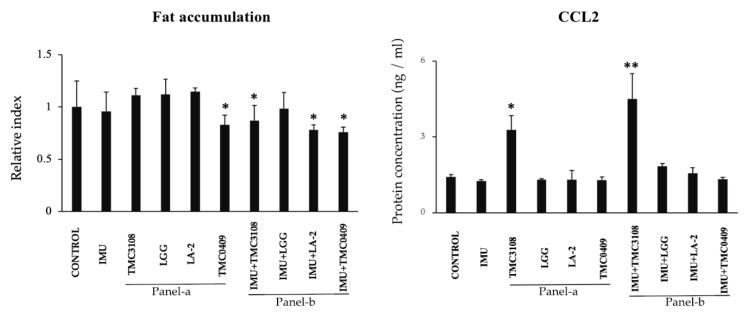
Immunobiotic mediated regulation of fat accumulation and inflammatory responses in an immune cell-adipocyte co-culture model. A total of 1 × 10^6^ immune cells/well of a 6-well plate were cultured on adipocytes 4-days differentiated. Bacterial samples (5 × 10^8^ cells/well) were also added and cultured for 2 or 4 days. Adipocyte cells were stained with Oil red O stained and fat accumulation in the adipocytes was quantitatively analyzed by image J software. The ELISA-based concentration of CCL2 protein in supernatant of adipocyte-culture stimulated by immune cells and lactic acid bacteria (LAB). All data shown are the mean ± SD of three independent experiments performed in triplicate. The asterisks: ‘*’ and ‘**’ indicated statistical differences either between bars of panel-a and control or between bars of panel-b and IMU, with significant levels of *p* < 0.05 and *p* < 0.01, respectively. Control, untreated adipocytes; IMU, cell free supernatant (CFS) medium obtained from immunocompetent cell (antigen presenting cell) culture; Panel-a, adipocytes directly exposed to LAB; Panel-b, adipocytes exposed to CFS obtained from different LAB treated immunocompetent cells; TMC3108, *Bifidobacterium bifidum* TMC3108; LGG, *Lactobacillus rhamnosus* GG; LA-2, *Lactobacillus rhamnosus* LA-2; TMC0409, *Lactobacillus paracasei* TMC0409.

Previously, we demonstrated that some immunobiotic strains are able to differentially modulate fat accumulation and proinflammatory mediators’ expression during short term adipogenic differentiation of PIP cells [16]. Then, we aimed to evaluate whether the same lactobacilli and bifidobacteria strains were able to modulate fat accumulation and CCL2 expression during LTAD. For this purpose, two sets of experiments were performed. First, porcine adipocytes were treated directly with bacterial strains. As shown in Figure 11 (Panel-a) no effect on fat accumulation was observed for the evaluated strains with the exception of *L. paracasei* TCM0409, which significantly reduced this parameter. In addition, only *B. bifidum* TCM3108 induced a significant up-regulation of CCL2 expression. In a second set of experiments, we performed several in vitro steps to simulate LAB-immune cells-adipocytes interaction. Antigen presenting cells isolated from porcine Peyer’s patches were stimulated with the different LAB strains and the cell free supernatants (CFS) were collected. Adipocytes were stimulated with the different CFS obtained in the previous step, and fat accumulation and CCL2 expression were evaluated. The CFS from immune cells stimulated with TCM3108, LA-2 and TCM0409 strains significantly reduced fat accumulation while only CFS from TCM3108 treatment improved CCL2 expression (Figure 11: Panel-b).

**Figure 12:**
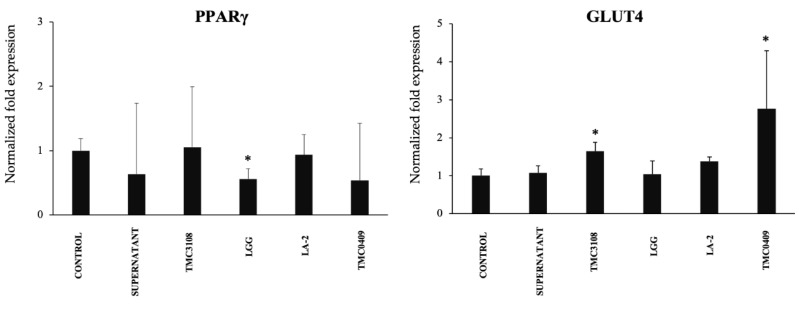
Immunobiotic mediated regulation of adipocyte differentiation in an immune cell-adipocyte co-culture model. The mRNA expressions of PPARγ and GLUT4 resulted from various immunobiotic lactic acid bacteria (LAB) stimulation into the co-culture model. A total of 1 × 10^6^ immune cells/well of 6-well plate were cultured on adipocytes 4-days differentiated. LAB samples (5 × 10^8^ cells/well) also added into the adipocyte plate and maintained for further 2 or 4 Days. All data shown are the average ±SD of 3 independent experiments performed in triplicate. The asterisk (*) indicated statistical differences when compared with control at the significance level of *p* < 0.05. TMC3108, *Bifidobacterium bifidum* TMC3108; LGG, *Lactobacillus rhamnosus* GG; LA-2, *Lactobacillus rhamnosus* LA-2; TMC0409, *Lactobacillus paracasei* TMC0409.

To gain further insight into the CFS mediated regulation of adipogenesis, the mRNA expression of PPARγ and GLUT4 were quantified. As shown in Figure 12, only the CFS from *L. rhamnosus* GG significantly down-regulated the expression of PPARγ, while TCM3108 and TCM0409 treatments up-regulated GLUT4 expression.

**Figure 13:**
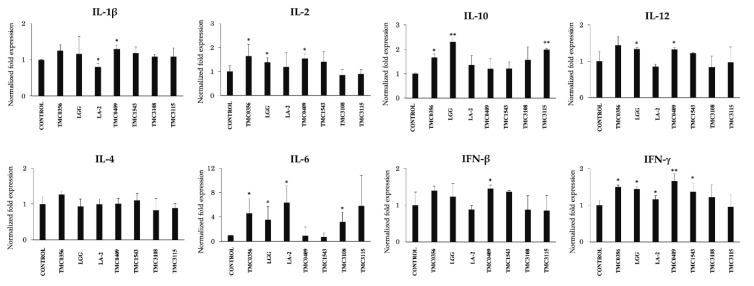
Immunobiotic mediated inflammatory responses in the antigen presenting cells (APCs) of Payer’s patches. The APCs were seeded at 1 × 10^6^ cells/well of 6-well plate and cultured with each bacterial strain (5 × 10^8^ cells/well) separately for 24 h and the mRNA expressions of IL-1β, IL-2, IL-4, IL-6, IL-10, IL-12, IFN-β and IFN-γ) were analyzed with RT-qPCR. Data shown are the mean ±SD of 3 independent experiments performed in triplicates. The asterisks: (*) and (**) indicated statistical differences with significant levels of *p* < 0.05 and *p* < 0.01, respectively. TMC0356, *Lactobacillus gasseri* TMC0356; LGG, *L. rhamnosus* GG; LA-2, *L. rhamnosus* LA-2; TMC0409, *L. paracasei* TMC0409; TMC1543, *Streptococcus thermophilus* TMC1543; TMC3108, *Bifidobacterium bifidum* TMC3108; TMC3115, *B. bifidum* TMC3115.

Finally, we aimed to characterize the cytokine profile induced by LAB strain on porcine antigen presenting cells that would be responsible for the effects on porcine adipocytes. A strain-dependent ability in the modulation of cytokine expression in porcine intestinal immune cells was observed (Figure 13). The most remarkable effect was found for *L. paracasei* TMC0409, which was able to significantly increase the expressions of *IL-1β*, *IL-2*, *IL-12*, *IFN-β* and *IFN-γ*. *L. rhamnosus* GG increased the mRNA expression levels of *IL-10*, *IL-12*, *IL-6* and *IFN-γ* (Figure 13). On the other hand, *L. rhamnosus* LA-2 significantly reduced the expression of *IL-1β* while it improved the levels of *IL-6* and *IFN-γ* (Figure 13). *Lactobacillus gasseri* TMC0356 stimulation increased the expression of *IL-2*, *IL-6*, *IL-10* and *IFN-γ* when compared with untreated control cells. *B. bifidum* TCM3115 enhanced the expression of *IL-6* and *IL-10* while *S. thermophilus* TCM1543 increased *IFN-β* and *IFN-γ* expression. *B. bifidum* TMC3108 only increased the expression of *IL-6* when compared to control cells (Figure 13).

## 4. Discussion

Adipocyte inflammation is a common feature of obesity and obesity-related complications. The inflammatory reactions in adipocytes initiate through their sensing of either microbial associated molecular patterns (MAMPs) or damage associated molecular patterns (DAMPs). Unlike the microorganism derived MAPMs which induce a rapid onset of proinflammatory reaction, DAMPs are endogenous molecules (e.g., free fatty acids, cholesterol, ceramides) derived from dead adipocytes, which can lead to a non-infectious chronic inflammation [37,38,39]. In our previous study, we have demonstrated the MAMPs-mediated acute inflammatory responses in adipocytes using a short-term differentiation period (4 days) of PIP cells [17,26,30]. However, like other standard in vitro adipocytes models, our previous approaches have the limitation of not able to address the mechanisms of sustained inflammation because of the short period of adipocytes culture. Therefore, here we expanded the utility and potential application of our in vitro PIP cells model by evaluating an LTAD period (20 days) in order to elucidate further insights of sustained inflammation on the pathobiology of adipocytes as well as to explore the prospects of beneficial immunomodulatory interventions for obesity.

The result of the present study indicated that cytosolic TG content of porcine mature adipocyte progressively increased over the period of LTAD evaluated here. This finding is consistent with previous studies demonstrating that in the mouse 3T3 adipocyte cell line, fat accumulation resulted from the increase of TG synthesis while cholesterol or its derivatives did not necessarily accumulate during the adipocyte differentiation [40]. The enlargement of lipid droplets and the enhancement of TG content are associated with an increase in lipolysis. High rate of lipolysis causes the increase of circulating FFA levels and their delivery to the liver increasing TG synthesis, which could lead to hepatic steatosis [41]. Along with TG synthesis in the liver, the increased delivery of FFA exacerbates insulin resistance, which promotes dyslipidemia. Saturated fatty acids, particularly lauric acid and palmitic acids, are capable of stimulating an inflammatory response through the TLR4 signaling pathway [42,43]. Lee et al. [44] evaluated the effect of different fatty acids on the TLR4 signaling and reported that lauric, palmitic, and stearic acids could induce COX-2 expression through an NF-kB-dependent mechanism in a macrophage cell line. These results suggest the existence of a correlation between fat accumulation and inflammatory responses in the adipocytes that experienced an LTAD.

In addition to fat accumulation, it was observed that LTAD led to an increased production of IL-6, IL-8 and specially CCL2 that was enhanced more than ten-fold in porcine adipocytes when compared to PIP cells. Both the mRNA and protein expression of CCL2 were significantly increased in adipocytes over the time of differentiation. The over-expression of CCL2 in adipose tissue increases macrophage recruitment [45], whereas a reduction of CCL2 or its receptor CCR2 lowered proinflammatory macrophages accumulation in the adipose tissue and provides protection from insulin resistance as well as hepatic steatosis [45]. Activated macrophages, both immigrant and adipose tissue residents, further trigger inflammatory responses through the release of various proinflammatory cytokines such as TNF-α and IL-6, via NF-kB activation and signaling (reviewed in [43]). In our hands, LTAD led to the production of new mature adipocytes, which will be able to secrete proinflammatory adipokines in response to the progressive stress of fat accumulation. These results suggest the existence of a correlation between fat accumulation and inflammatory responses in porcine adipocytes during LTAD. This is in line with previous studies reporting the correlation of fat accumulation and the increased expression of cytokines and chemokines including CCL2, IL-6 and IL-8 in the obese individuals [46].

Adiponectin and leptin are two physiologically important adipokines released from white adipocytes [46,47]. In addition to fat accumulation dynamics, we observed here that adiponectin and leptin production followed a moderate increasing trend over LTAD. The secretion of adiponectin and leptin is known to involve distinct intracellular trafficking pathways [47], suggesting that adipocytes rely on different avenues for the constitutive and regulated secretion of these adipokines. It has been reported that adipocyte size correlated with the production of adiponectin and leptin [3,8]. Then, it is tempting to speculate that the continuous differentiation of PIP cells during LTAD led to the development of a higher number of matured adipocytes that upon reaching a critical mass of fat accumulation increased their production of adiponectin and leptin. In addition to metabolic and endocrine functions, adipocytes derived from the differentiation of porcine intramuscular preadipocytes could be a source for adult stem cells. It was reported that human adipocytes generated through adipogenic differentiation in vitro exhibit similar physiological properties of bone marrow-derived mesenchymal stem cells [48] because of the more abundant cell number and the high purity as compared to adipose-derived stem cells [49]. A recent study demonstrated that porcine-derived differentiated fat cells exhibit efficient proliferative activity, multipotent differentiation to adipocytes, osteoblasts, and myocytes, normal diploid karyotypes during the long-term in vitro culture [50]. Therefore, pigs being considered as the closest approximate animal model for studying human disease, porcine derived differentiated adipocytes maintained in long-term in vitro culture can be an excellent stem cell model applied to tissue engineering and regenerative biomedicine.

Of note, the changes in the levels of adipokines observed during the LTAD evaluated here can be explained by other processes that are not mutually exclusive. On the one hand, the increased synthesis of CCL2, IL-6, IL-8, adiponectin and leptin during LTAD of PIP cells could be associated to the release of DAMPs. The progressive death of adipocytes may lead to the release of DAMPs, which may subsequently induce a low-grade inflammatory response. On the other hand, the long-term culture of adipocyte progenitors such as preadipocytes can induce senescence of the growing cells, which function as mediators of inflammation associated with obesity [51,52]. It was reported that in addition to no longer undertaking their original function effectively, senescent cells can induce the secretion of proinflammatory factors in the tissue in which they reside [53]. Cellular senescence in adipocytes attenuates the physiological resolution of inflammation leading to a low-grade chronic inflammation, insulin resistance, type-2 diabetes, and companion senescence-associated secretory phenotype in the obese individual [51,52,53], indicating that these phenotypes are associated with impaired adipokine production. A detailed evaluation of senescence, cell death and DAMPs-mediated signaling pathway in PIP cells during LTAD would be of great value to further characterize this process in vitro and establish in parallel with the in vivo physiology.

We also evaluated the influence of TLRs activation on the degree of fat accumulation and cytokines production by porcine adipocytes during LTAD. Results demonstrated that fat accumulation in adipocytes was increased after stimulation with poly(I:C) or LPS which induced the activation of TLR3 and TLR4, respectively. We previously reported the presence of TLR1-9 in PIP cells [17], and in a follow up study, we demonstrated the activation of TLR2, TLR3 and TLR4 in differentiated adipocytes after specific ligand stimulation [26]. Studies have demonstrated that TLR2 and TLR4 agonists activate macrophages and adipocytes in the adipose tissue, resulting in increased release of various adipokines [54,55]. Fat accumulation and obesity-induced activation of TLR-mediated proinflammatory signaling cascades were demonstrated in the adipose tissue of mice [56]. Kim et al. [56] provided evidence of increased expression of TLR 1-9 and TLR 11-13 in murine adipose tissue in response to obesity induced by high-fat diet administration, causing activation of both MyD88- and non-MyD88 signaling cascades as well as downstream up-regulation of NF-kB activity and the subsequent release of adipokines.

The expression patterns of adipokines varied significantly with the timing of TLR-ligands stimulation. The expression of CCL2, IL-6 and IL-8 were increased in the later time points of stimulation when a decreased IL-10 expression was noticed. Interestingly, cytokine expression dynamics varied between ligands. Our results are in accordance with a study conducted by Kopp et al. [24] who have demonstrated that the specific ligands of TLRs can influence IL-6 and CCL2 release in human adipocytes in a ligand-specific manner. The stimulation with poly(I:C) resulted in CCL2 production at an early stage of the study while the other ligands triggered the synthesis of this chemokine at later stages. TLR3 is expressed in the porcine adipocytes [26] as well as human adipocytes [57]. TLR3 can recognize mRNA from dying cells, a process frequently observed in obese adipose tissue [55,57]. It was reported that the in vitro stimulation of human and mouse adipocytes with poly(I:C) led to an increased expression of proinflammatory factors such as IL-6, IL-8 and CCL2. However, TLR3 signaling had no influence on the obesity-induced inflammation in adipose tissue in vivo [57]. On the other hand, LPS and TNF-α stimulation led to a strong induction of CCL2 protein at a later stage of the differentiation period. The TNF-α-mediated expression of IL-6 and IL-8 was stronger than the TLRs ligand stimulations.

In addition, we evaluated here the ability of LAB to modify the fat accumulation and CCL2 expression of 20-day-old porcine adipocytes through host’ intestinal immune-competent cells. For this purpose, we stimulated porcine APCs from Peyer’s patches with different LAB strains, collected the CFS and treated porcine adipocytes with them. Results indicated that the rate of cytosolic fat accumulation was significantly reduced in a strain dependent manner. Remarkably, a reduced fat accumulation was recorded when the CFS from *L. rhamnosus* LA-2 and *L. casei* TMC0409-treated immune cells were used. In addition, no significant effect in the expression of CCL2 was observed after the treatment of adipocytes with CFS with the exception of CFS from the TMC3108 strain that up-regulated this chemokine. These results contrast significantly with our previous studies on four-day-old porcine adipocytes. When the CFS from *L. rhamnosus* LA-2 and *L. rhamnosus* GG were used to stimulate four-day-old porcine adipocytes, a significant reduction in the expression of CCL2 was observed, even after the stimulation with TNF-α [17]. Those previous findings were in agreement with studies performed in the murine macrophage-like cell line J774.1 stimulated with *L. rhamnosus* GG followed by a co-culture with the mouse preadipocyte cell line 3T3-L1, which resulted in a decreased fat accumulation [58]. The effect of the GG strain was associated to its ability to improve IFN-γ in immune cells and reduced PPAR-γ mRNA expression in 3T3-L1 cells [59,60]. Here, we demonstrated that *L. rhamnosus* GG was capable of upregulating the expression of IFN-γ in porcine APCs as well as down regulating PPAR-γ in adipocytes. However, the GG strain was not capable of reducing fat accumulation or CCL2 expression. These results show an important difference between the 4- and 20-day-old porcine adipocytes in terms of the most efficient immunobiotic strain capable of regulating their response.

Interestingly, *L. rhamnosus* GG, similarly to the strains able to reduce fat accumulation in 20-day-old porcine adipocytes, increased the expression of IFN-γ, IL-6, IL-12 and IL-10 in APCs. Then, it is tempting to speculate that the presence of these cytokines in CFS would not be responsible for modulating fat accumulation in adipocytes. On the contrary, the GG strain was not capable of modulating IL-1β or IFN-β expression in immune cells as the strains with the ability to reduce fat accumulation. It has been shown that IL-1β impairs insulin sensitivity in adipose tissue by affecting insulin signal transduction [61]. Paradoxically, it was reported that adipogenesis and the expression of the markers *PPARγ*, *CEPBA*, *FABP4*, and *GLUT4* were significantly elevated in IL-1β^−/−^ mice under a high-fat diet treatment [62]. Moreover, it was shown that IL-1Ra^−/−^ mice have a lean phenotype due to decreased fat mass, related to a defect in adipogenesis and increased energy expenditure [63]. On the other hand, it was demonstrated that the overexpression of IFN-β blocked body weight gain and adipose tissue expansion in mice under high-fat diet treatment. Furthermore, IFN-β protected animals against adipose tissue inflammation [64]. Then, our results indicate that the cytokines able to regulate fat accumulation and inflammation in porcine adipocytes are different when 4- and 20-day-old cells are considered. Evaluating the effects of IFN-γ, IL-1β and IFN-β signaling pathways in PIP cells and in 4- and 20-day-old porcine adipocytes in terms of their fat accumulation abilities and the inflammatory cytokines expression will significantly enhance our understanding of the biology of these porcine cells. In addition, those studies could improve our knowledge of the effect of immunobiotics and will enhance our capacity to select the most appropriate strains for their application in the prevention of obesity and its related inflammatory complications.

## 5. Conclusions

We presented here a long-term adipogenic differentiation model using a porcine intramuscular preadipocytes cell line (PIP cells) and evaluated the effects of the sustained inflammation induced by TNF-α and TLRs ligands on the degree of fat accumulation and adipokine production. In our hands, activation of TNF, TLR3 and TLR4 pathways resulted in an increased fat accumulation and inflammatory factors expression of the adipocytes. Since in obese subjects, adipocytes are subject to sustained inflammatory responses, the characterization of the immunobiology and adipogenesis of PIP cells under a sustained rather than an acute inflammatory environment performed in this work indicate that this cell line could be used for the study of metabolic and immunological alterations in adipocytes in the context of obesity and the potential therapeutic alternatives to mitigate those effects. Despite the need for more in-depth investigations of immune signaling pathways, metabolic activities, senescence and cell death to establish conclusively a physiological relevance of LTAD in PIP cells to in vivo human adult intramuscular adipocytes, this is a first step in the positioning of this cell line as a useful laboratory tool for obesity studies.
